# EMP3 as a prognostic biomarker correlates with EMT in GBM

**DOI:** 10.1186/s12885-023-11796-0

**Published:** 2024-01-17

**Authors:** Li Li, Siyu Xia, Zitong Zhao, Lili Deng, Hanbing Wang, Dongbo Yang, Yizhou Hu, Jingjing Ji, Dayong Huang, Tao Xin

**Affiliations:** 1 Department of Oncology, the Second Affiliated Hospital of Harbin Medical University, Harbin, 150081 China; 2 Department of Oncology, The Beidahuang Group General Hospital, Harbin, 150006 China; 3 Department of Anesthesiology and Pain Rehabilitation, School of Medicine, Shanghai YangZhi Rehabilitation Hospital (Shanghai Sunshine Rehabilitation Center), Tongji University, Shanghai, 201619 China; 4 Department of Neurosurgery, the Second Affiliated Hospital of Harbin Medical University, Harbin, 150081 China; 5 Division of Molecular Neurobiology, Department of Medical Biochemistry and Biophysics, Karolinska Institutet, Stockholm, Sweden; 6 Department of Pathology, the Second Affiliated Hospital of Harbin Medical University, Harbin, 150081 China

**Keywords:** Glioblastoma, Epithelial membrane protein 3, Epithelial-mesenchymal transition, Prognostic biomarker

## Abstract

**Background:**

Glioblastoma (GBM) is the most aggressive malignant central nervous system tumor with a poor prognosis.The malignant transformation of glioma cells via epithelial-mesenchymal transition (EMT) has been observed as a main obstacle for glioblastoma treatment. Epithelial membrane protein 3 (*EMP3*) is significantly associated with the malignancy of GBM and the prognosis of patients. Therefore, exploring the possible mechanisms by which *EMP3* promotes the growth of GBM has important implications for the treatment of GBM.

**Methods:**

We performed enrichment and correlation analysis in 5 single-cell RNA sequencing datasets. Differential expression of *EMP3* in gliomas, Kaplan–Meier survival curves, diagnostic accuracy and prognostic prediction were analyzed by bioinformatics in the China Glioma Genome Atlas (CGGA) database and The Cancer Genome Atlas (TCGA) database. *EMP3*-silenced U87 and U251 cell lines were obtained by transient transfection with siRNA. The effect of *EMP3* on glioblastoma proliferation was examined using the CCK-8 assay. Transwell migration assay and wound healing assay were used to assess the effect of EMP3 on glioblastoma migration. Finally, quantitative real-time polymerase chain reaction (qRT-PCR) and western blot were used to detect the mRNA and protein expression levels of EMT-related transcription factors and mesenchymal markers.

**Results:**

*EMP3* is a EMT associated gene in multiple types of malignant cancer and in high-grade glioblastoma. *EMP3* is enriched in high-grade gliomas and isocitrate dehydrogenase (IDH) wild-type gliomas.*EMP3* can be used as a specific biomarker for diagnosing glioma patients. It is also an independent prognostic factor for glioma patients' overall survival (OS). In addition, silencing *EMP3* reduces the proliferation and migration of glioblastoma cells. Mechanistically, *EMP3* enhances the malignant potential of tumor cells by promoting EMT.

**Conclusion:**

*EMP3* promotes the proliferation and migration of GBM cells, and the mechanism may be related to *EMP3* promoting the EMT process in GBM; *EMP3* may be an independent prognostic factor in GBM.

**Supplementary Information:**

The online version contains supplementary material available at 10.1186/s12885-023-11796-0.

## Introduction

GBM is the most common and lethal primary brain tumor in adults [1], accounting for 12–15% of intracranial tumors statistically [2]. GBM is highly aggressive with an abysmal prognosis. Despite employing a combination of surgery, radiotherapy, chemotherapy, and immunotherapy, the survival rate remains dishearteningly low, with fewer than 30% of patients surviving past two years [3]. Clinical diagnosis and treatment of GBM are challenging due to its aggressive nature, genetic complexity, and our limited understanding of the underlying disease mechanisms.

*Epithelial membrane protein 3 (EMP3)* belongs to the Peripheral Myelin Protein 22-kDa (PMP22) gene family. This molecular family plays a crucial role in regulating cell growth and orchestrating tissue-specific processes like chondrocyte differentiation and angiogenesis [4, 5]. *EMP3* is located on chromosome 19q13.3 [6], encodes 163 amino acids at 18 kDa, contains four transmembrane structural domains and two n-linked glycosylation sites on the first extracellular loop [7, 8], and is involved in cell proliferation, differentiation, apoptosis and migration [9].

*EMP3* was recently reported to be a tumor suppressor gene for several solid tumors, and is drawing attention as a novel prognostic marker [10], such as glioma [6], esophageal squamous cell carcinoma [11], colorectal cancer [12], and non-small cell lung cancer [13]. In some kinds of malignancies like breast cancer, and hepatocellular carcinoma, *EMP3* can act as an oncogene [14, 15]. Increasing evidence suggests that *EMP3* might be a driving force in tumorigenesis and the progression of certain cancers. Low *EMP3* expression has been linked to inhibiting the progression of gastric cancer, notably curtailing the migration and invasion of cancer cells [16]. Interestingly, *EMP3* expression is found to be altered in glioma tissue, but the gene expression status and biological function of *EMP3* in glioblastoma remains unknown and unclear. Epithelial-to-mesenchymal transition (EMT) is the process by which epithelial cells acquire a mesenchymal stem cell phenotype. Notably, a large body of literature has demonstrated the involvement of EMT in the metastasis of tumor cells [17, 18].

In this study, we conducted an in-depth exploration of the clinicopathological characteristics, prognostic implications, and diagnostic potential of *EMP3* in glioblastoma patients, employing both bulk and single-cell analysis methods. Our experimental observations revealed the pivotal role of *EMP3* in amplifying the malignant progression of GBM. Our findings suggest that *EMP3* expression may amplify the aggressive trajectory of GBM via regulation of the epithelial-mesenchymal transition (EMT) process. These insights indicate potential advantages of therapeutically targeting *EMP3* and modulating the EMT process.

## Material and method

### Bioinformatics analysis

The data in this study were obtained from the CGGA database (http://www.cgga.org.cn) and TCGA database (https://portal.gdc.cancer.gov/).The expression of *EMP3* in different clinicopathological types of gliomas was analyzed. Based on the median expression levels of *EMP3*, we analyzed the survival data of glioma patients, ROC curves were used to measure the diagnostic value of *EMP3* in glioma patients, and univariate and multifactorial analyses of the prognostic role of *EMP3* in glioma were performed using COX regression. We then developed nomogram models to analyze and assess the predictive value of *EMP3* expression in glioma patients at 1, 3 and 5 years of overall survival, and calibration curves were plotted at 1, 3 and 5 years to evaluate the performance of the nomogram.

### Cell lines and cell culture

Human glioblastoma cells U87MG and U251MG (purchased from the Cell Bank of the Chinese Academy of Sciences, Shanghai, China) were cultured in DMEM high sugar medium supplemented with 10% fetal bovine serum (FBS, Every Green, China) and 1% double antibody: penicillin–streptomycin (Biyuntian, Shanghai, China), and then cultured at the atmosphere of 5% CO2 in 37 ◦C constant temperature incubator.

### Quantitative reverse- transcription polymerase chain reaction (qRT- PCR)

According to the manufacturer’s protocol, total RNA was extracted using the TRIzol reagent (Seven, China). The purity and concentration of total RNA extracted from the cells were then measured by UV spectrophotometer (Nikon, Japan), and the corresponding mRNAs we needed were amplified by reverse transcription. All qPCR reactions were performed by a real-time fluorescent quantitative PCR instrument (BIO-RAD, USA), and the expression levels of the target genes were analyzed. Primers for the target genes: VIM, FOS, SNAI2 and TWIST1 were designed by Beijing Rui Bo Xing Ke Biotechnology Co Ltd, and the primer sequences are shown in Table 1. The expression levels of *EMP3* were calculated using the 2^ − △△CT method. *EMP3* expression levels were normalized to those of GAPDH.

**Table 1:** Part of primer sequences

Gene	Sequences
VIM	5’-GACGCCATCAACACCGAGTT-3’
FOS	5’-GAAGACCGAGCCCTTTGAT-3’
SNAI2	5’-TGTGACAAGGAATATGTGAGCC-3’
TWIST1	5’-GTCCGCAGTCTTACGAGGAG-3’
GAPDH	5’-UGACCUCAACUACAUGGUUTT-3’

### Western Blotting (WB) analysis

Protein was extracted from the cells were resolved by SDS-PAGE and then transferred to PVDF membranes (IPVH00010, Millipore), and then incubated with primary antibodies diluted in blocking buffer at 4 °C overnight. The following primary antibodies were used: Mouse Anti-β-Actin (HC201, TransGen Biotech, 1/2000), HRP conjugated Goat Anti-Mouse IgG (H + L) (GB23301, Servicebio, 1/2000), Rabbit Anti EMP3 (DF14661, Affinity, 1/1000), Rabbit Anti TWIST1(AF4009, Affinity, 1/1000), Rabbit Anti SNAI2 (PB9439, Boster, 1/1000), Rabbit Anti FOS (AF5354, Affinity, 1/1000), Mouse Anti-VIM (60330–1-Ig, Proteintech,1/10000), HRP conjugated Goat Anti-Rabbit IgG (H + L) (GB23303, Servicebio, 1/2000), HRP conjugated Goat Anti-Mouse IgG (H + L) (GB23301, Servicebio, 1/2000)was used. Finally, the antigen–antibody reaction was visualized by the enhanced Pierce ECL Western blotting substrate kit (Thermo Scientific/ Pierce, Rockford, IL, USA).

### Transient transfection

The negative control (NC), *si-EMP3-1, si-EMP3-2, si-EMP3-3* and GAPDH were purchased from Suzhou Jima Biotechnology Co. U87 cells and U251 cells at logarithmic growth were collected 24 h before transfection. Cells were cultured in 6-well plates (1–2 × 105 cells/well) at 80–90% concentration. A mixture of liposome 2000, siRNA and serum-free DMEM was added to the wells for transfection according to the manufacturer's instructions and replaced with a serum-containing medium 6–10 h later. The sequences of these siRNAs are listed in Table 2.

**Table 2:** Part of primer sequences

Gene	Sequences
NC	5’-CCTGAATCTCTGGTACGACTGC-3’
si-EMP3-1	5’-GCAGUAAUGUCAGCGAGAATT-3’
si-EMP3-2	5’-GUCUCUCCUUCAUCCUGUUTT-3’
si-EMP3-3	5’-GUCAGCGGCAUCAUCUACATT-3’
GAPDH	5’-UGACCUCAACUACAUGGUUTT-3’

### CCK-8 assay

Cells from the U87MG and U251MG control and *si-EMP3* groups were inoculated in 96-well plates. Cell counting kit-8 (Dojindo Laboratories, Japan) was diluted to 10% using DMEM and added to the wells to measure the OD value at 450 nm using a microplate reader.

### Transwell assays

Five thousand cells were inoculated into the upper chamber of the 24-well transwell chamber and cultured in a serum-free medium for 24 h. At the same time, 600 µL of complete medium was added to the lower chamber. The cells in the upper and lower chambers were then fixed in 4% paraformaldehyde for 30 min and stained with 0.1% crystal violet solution for 30 min. The upper chambers were washed with PBS, and the cells were counted by 200 × light microscopy.

### Wound healing assays

The complete medium was removed from the 6-well plates and replaced with a serum-free medium. A 200uL gun was used to draw a uniform, straight line vertically, and PBS was washed three times to remove floating cells. Images of 0h and 24h scratches were recorded by microscopic photography.

### Statistical analysis

Statistical analyses and visualization were performed in R (version 3.6.3),IBM SPSS Statistics (version 25.0), and GraphPad Prism (version 7.0.0). All data are expressed as mean ± standard error of the mean. Student's t-test or one-way ANOVA was used to compare the differences among multiple groups. Statistical significance was set at *P* < 0.05.

## Results

### *EMP3* is a EMT associated gene in multiple types of malignant cancer and in high-grade glioblastoma, but not in low-grade glioma

**Fig. 1:**
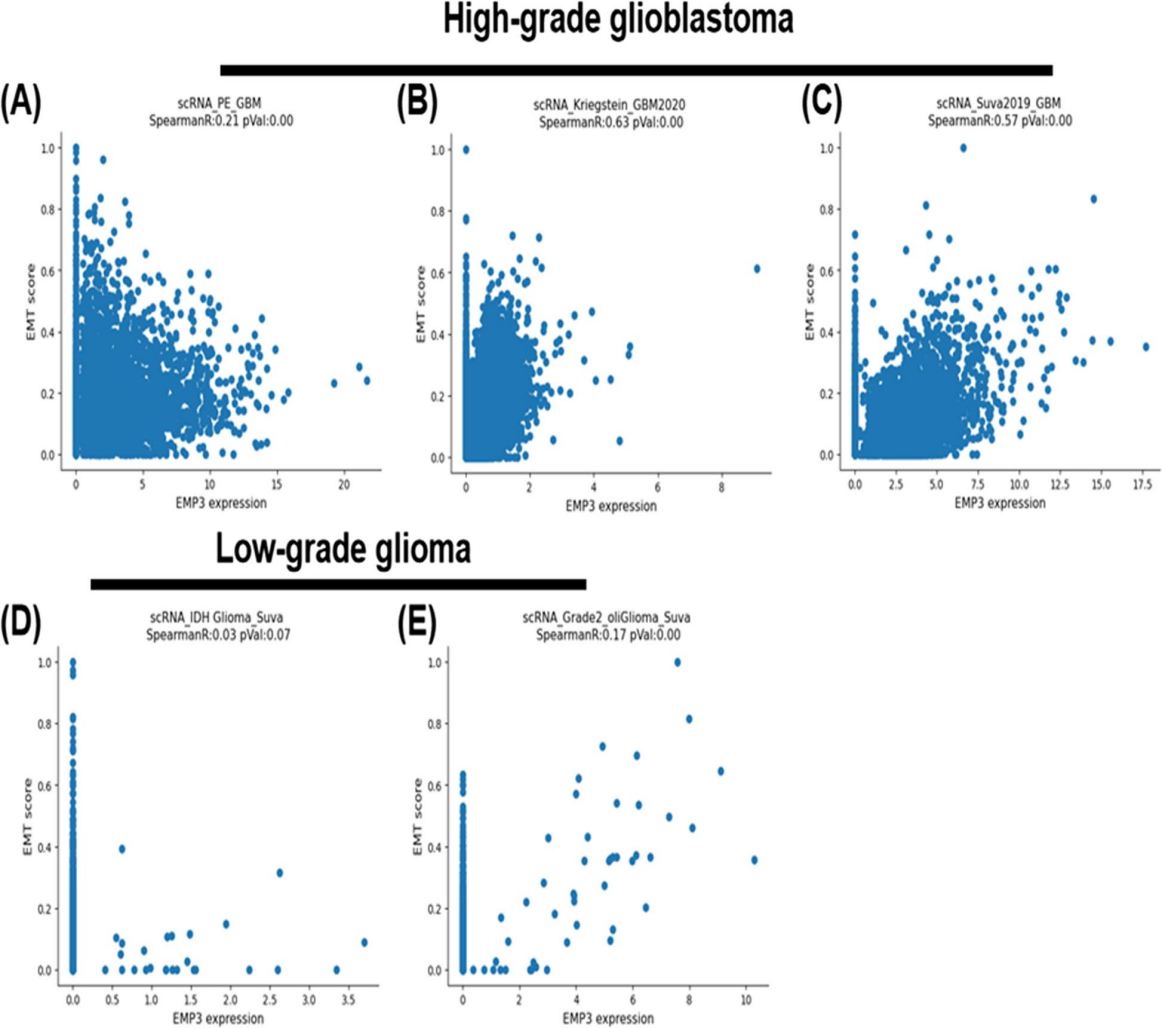
Scatter plot representing the correlation between *EMP3* expression and EMT score in individual tumor cells from three high-grade glioblastoma datasets (**A-C**) and two low-grade glioma datasets (**D-E)**. Each dot represents a single cell. The name of the dataset, along with the correlation R value and p value, are specified at the top of each plot

In our previous study, we used the TCGA database to collect mRNA transcriptome sequencing data from 11,065 samples across 33 distinct cancer types [19]. Our main goal was to evaluate the epithelial-mesenchymal transition (EMT) for each cancer type using enrichment analysis. To identify nonlinear associations, we applied pairwise mutual information to assess the relationship between each gene and the EMT score. This approach led us to find 1464 genes significantly associated with EMT in at least 22 cancer types (66.7% of cancer types). To further examine EMT, we analyzed the EMT score in single-cell RNA sequencing data from three glioblastoma datasets and two low-grade glioma datasets [20], resulting in the identification of 47 genes consistently associated with EMT across all three high-grade glioblastoma datasets (Supplementary Table 1, 2 and 3). Comparing 1464 EMT-associated genes in glioblastoma with the pan-cancer dataset, we uncovered three key genes: *VIM, EMP3, and AHNAK*. Interestingly, *EMP3* demonstrated a unique connection with mesenchymal transition specifically in aggressive glioblastoma (Fig. 1A-C), while not showing a similar association in low-grade oligodendroglioma or IDH-mutant astrocytoma (Fig. 1D-E, Supplementary Table 4 & 5) [21, 22].

### Expression of *EMP3* is enriched in gliomas and correlates closely with multiple clinicopathological parameter in glioma patients

**Fig. 2:**
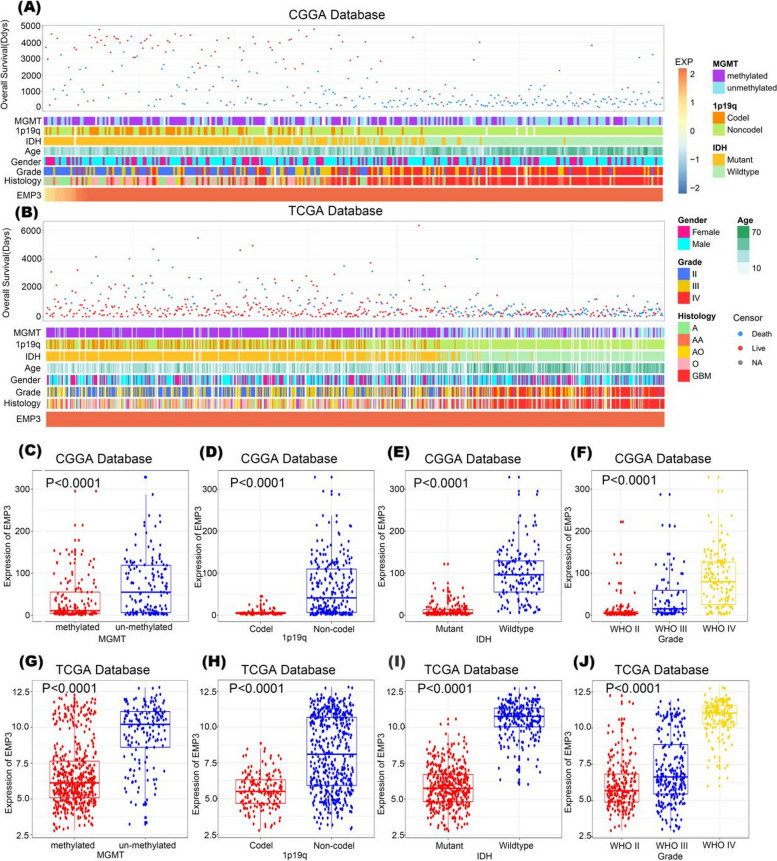
Association between *EMP3* and clinicopathological characteristics of gliomas (**A**). The landscape of *EMP3*- related clinicopathological features of gliomas in the Chinese Glioma Genome Atlas (CGGA) database (**B**). The landscape of *EMP3*- related clinicopathological features of gliomas in The Cancer Genome Atlas (TCGA) database (**C**) and (**G**). *EMP3* was significantly increased in the O6- methylguanine- DNA methyltransferase (MGMT) promoter–unmethylated gliomas in the CGGA and TCGA databases. The significance of the difference was tested using an unpaired t test (**D**) and (**H**). *EMP3* was significantly increased in gliomas without 1p19q codeletion in the CGGA and TCGA databases. The significance of the difference was tested with an unpaired t test (**E**) and (**I**). *EMP3* has significantly increased in gliomas without isocitrate dehydrogenase (IDH) mutation in the CGGA and TCGA databases. The significance of the difference was tested with an unpaired t test (**F**) and (**J**). *EMP3* was significantly increased in higher-grade gliomas in the CGGA and TCGA databases. One-way ANOVA tested the significance of the difference

To explore the differences in *EMP3* mRNA expression in glioma patients with different clinical characteristics, data extracted from the TCGA database (*n* = 702) and CGGA (*n* = 325) was mined and compared. *EMP3*, *MGMT* promoter methylation status, 1p19q co-deletion status, *IDH* mutation status, and WHO grade showed asymmetric distributions in the CGGA and TCGA datasets (Fig. 2A, B). Through comparative analysis of these groups, in the CGGA database, *EMP3* was highly enriched in higher-grade gliomas (*P* < 0.0001, Fig. 2C) and *IDH*-wildtype gliomas (*P* < 0.0001, Fig. 2D). Moreover, *EMP3* showed high expression in samples without 1p19q co-deletion (*P* < 0.0001, Fig. 2E) and in samples witho ut *MGMT* promoter methylation (*P* < 0.0001, Fig. 2F). The above results were validated in the TCGA database (*P* < 0.0001, Fig. 1G-J). These results suggested that *EMP3* was abundantly expressed in more malignant gliomas.

### *EMP3* is associated with the prognosis of glioma patients

**Fig. 3:**
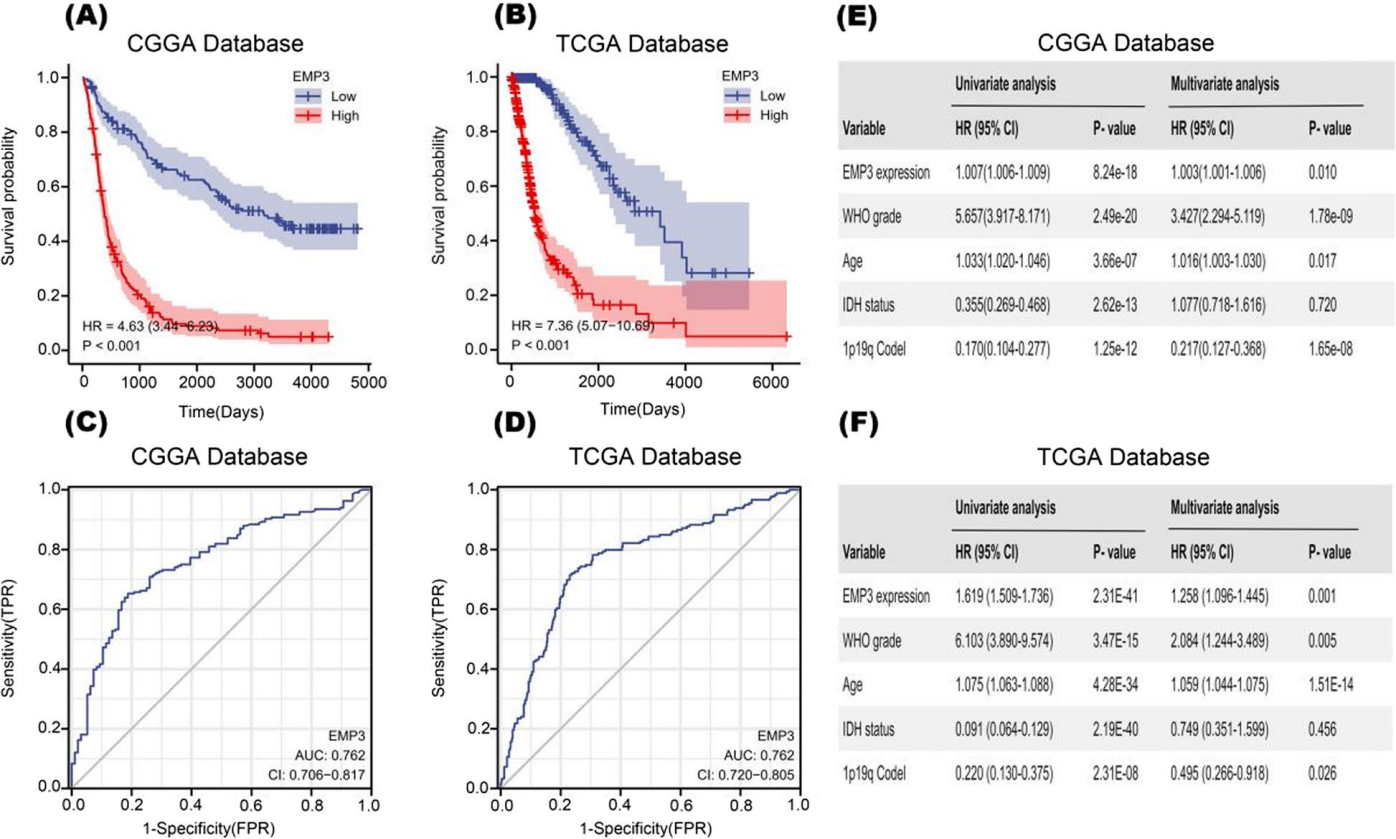
*EMP3* is a biomarker of gliomas with prognostic significance (**A**) and (**B**). Kaplan–Meier curves show that different expression levels of *EMP3* in the CGGA and TCGA databases correlate with survival in glioma patients (**C**) and (**D**). ROC curve analysis: accuracy of *EMP3* for diagnosing glioma patients in the CGGA and TCGA databases (**E**) and (**F**). Univariate and multivariate analysis of prognostic parameters in the Chinese Glioma Genome Atlas (CGGA) database and The Cancer Genome Atlas (TCGA) database overall survival (OS) P-value is listed in the gram

To investigate the prognostic, predictive value of *EMP3* in glioma patients, we performed Kaplan- Meier and ROC curves and Cox proportional risk models based on the CGGA and TCGA databases. Analysis of glioma patients collected in the CGGA database showed that the overall survival of patients with high *EMP3* expression was significantly shorter than that of patients with low *EMP3* expression (*P* < 0.001, HR:4.63, Fig. 3A), and the same results were obtained in the TCGA database (*P* < 0.001, HR:7.36, Fig. 3B). As indicated by the area under the ROC curve, *EMP3* showed high diagnostic accuracy for patients with glioma (CGGA *EMP3*:AUC = 0.762, 95% CI:0.706–0-0.817; TCGA *EMP3*:AUC = 0.762, 95% CI:0.720–0-0.805, Fig. 3C, D). In Cox regression analyses, *EMP3* was an independent prognostic factor in the CGGA and TCGA databases, and by univariate and multifactorial analyses including WHO classification, age at diagnosis, *IDH* mutation status and 1p19q co-deletion these findings showed that abnormal *EMP3* expression predicted poor prognosis in gliomas (Fig. 3E, F).

**Fig. 4:**
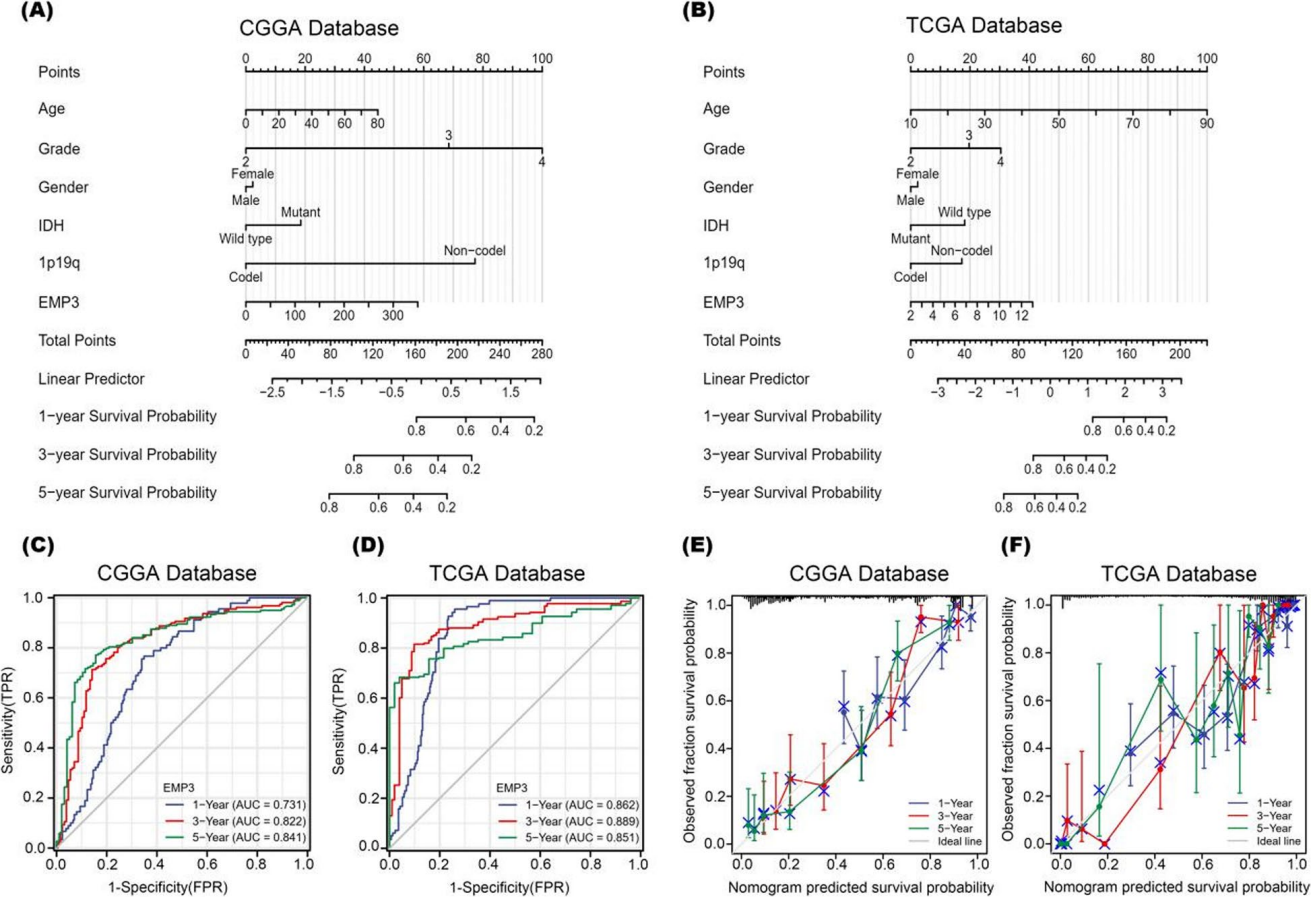
Individualized predictive models for patients with glioblastoma (**A**) and (**B**).The nomogram for predicting 1-, 3- and 5-year overall survival probabilities for glioma patients based on the CGGA and TCGA databases (**C**) and (**D**). ROC curve analysis: the accuracy of *EMP3* in predicting the prognosis of glioma patients at 1, 3 and 5 years in the CGGA and TCGA databases (**E**) and (**F**). Calibration curves for predicting 1-, 3- and 5-year overall survival probabilities for glioma patients using the nomogram method based on the CGGA and TCGA databases

Finally, we constructed individualized prediction models to facilitate the clinical application of prognostic prediction models. Based on the CGGA and TCGA databases, independent predictors such as WHO grade, age, *IDH* mutation status, 1p19q co-deletion, and *EMP3* expression level were incorporated into the prognostic model as shown in Fig. 4A, B, to predict the survival of glioma patients. The AUCs for 1, 3, and 5-year overall survival of glioma patients based on the CGGA database were 0.731, 0.822 and 0.841, respectively (Fig. 4C). The AUCs for 1, 3, and 5-year overall survival of glioma patients based on the TCGA database were 0.862, 0.889 and 0.851, respectively (Fig. 4D). Calibration plots were created to show the degree of agreement between observed and predicted survival outcomes for the 1-, 3- and 5-year prognostic models. The calibration plots showed the best agreement (consistency indices were: CGGA: 0.759, 95% CI: 0.743–0.775; TCGA: 0.865, 95% CI: 0.852–0.877, Fig. 4E, F), indicating that the prognostic models we constructed had good predictive power.

Therefore, *EMP3* is a reliable biological marker for predicting the prognosis of patients with glioma.

### *EMP3* affects the malignant behavior of glioblastoma

**Fig. 5:**
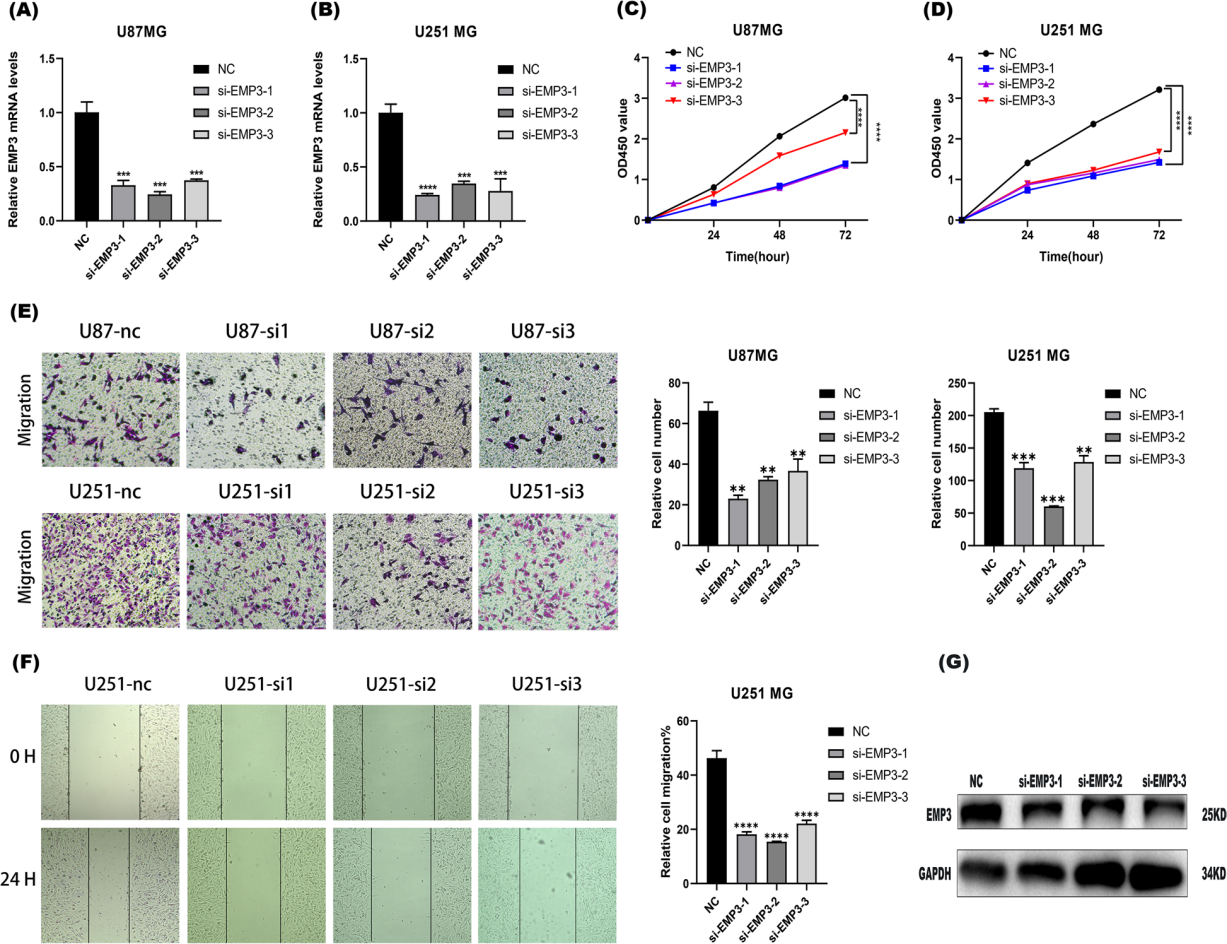
Knockdown of *EMP3* inhibits the malignant phenotypes of glioblastomas (**A**) and (**B**). The expression of *EMP3* was significantly reduced after transfection of U87 and U251 cells for 72 h cultured with siRNA, and GAPDH was used as an internal control (**C**) and (**D**). The proliferation rate of U87 and U251 cells in the *si-EMP3* group was significantly lower than that of the NC group at 24 h, 48 h, 72 h (**E**). The number of migrating U87 and U251 cells in the *si-EMP3* group was lower than that in the NC group at 72-96 h (**F**). Wound healing results indicated the migration capacity of the si-NRP1 group was significantly lower than that of the NC group at 72-96 h.The knockdown efficiency of *EMP3* in U251 glioma cells are detected by western blot (**G**). Cells were divided into four groups, NC (negtive control) group, siRNA *EMP3-1*, siRNA *EMP3-2* and siRNA *EMP3-3* group. U251 cells were lysis after siRNA incubation for 72 h. All of the full-length blots/gels of WB are presented in Supplementary Figure 1A and B. *p < 0.05, **P < 0.01, ***P < 0.001, ****p < 0.0001

The role of *EMP3* in different malignancies is controversial. To determine the biological function of *EMP3* in glioblastoma, we knocked down *EMP3* in two glioblastoma cell lines (U87 MG and U251 MG) by transient transfection with siRNA (*si-EMP3-1,si-EMP3-2 and si-EMP3-3*) for 72 h cultured with siRNA. The expression levels of *EMP3* mRNA were then quantified by qPCR (*p* < 0.001, Fig. 5A, B). After transient transfection, cell proliferation capacity was significantly inhibited in the *si-EMP3* group compared to the NC group at 24 h, 48 h, 72 h (*p* < 0.0001, Fig. 5C, D). In addition, silencing *EMP3* at 72-96 h significantly reduced the number of tumor cells that migrated (*p* < 0.01, Fig. 5E). Similarly, after 24 h of incubation in a serum-free medium for the U251 cell line, Wound healing results indicated that the migratory capacity of tumor cells in the knock-down group was significantly inhibited compared to cells in the NC group at 72-96 h (*p* < 0.0001, Fig. 5F). The knockdown efficiency of EMP3 in glioma cells are detected by western blotting experiments (*p* < 0.01, Fig. 5G). All of the full-length blots/gels are presented in Supplementary Figure 1A and B. Thus, it can be inferred that *EMP3* acts as an oncogene in glioblastoma, promoting the proliferation, migration of tumor cells.

### *EMP3* affects the malignant phenotype of glioblastoma by promoting the EMT process

**Fig. 6:**
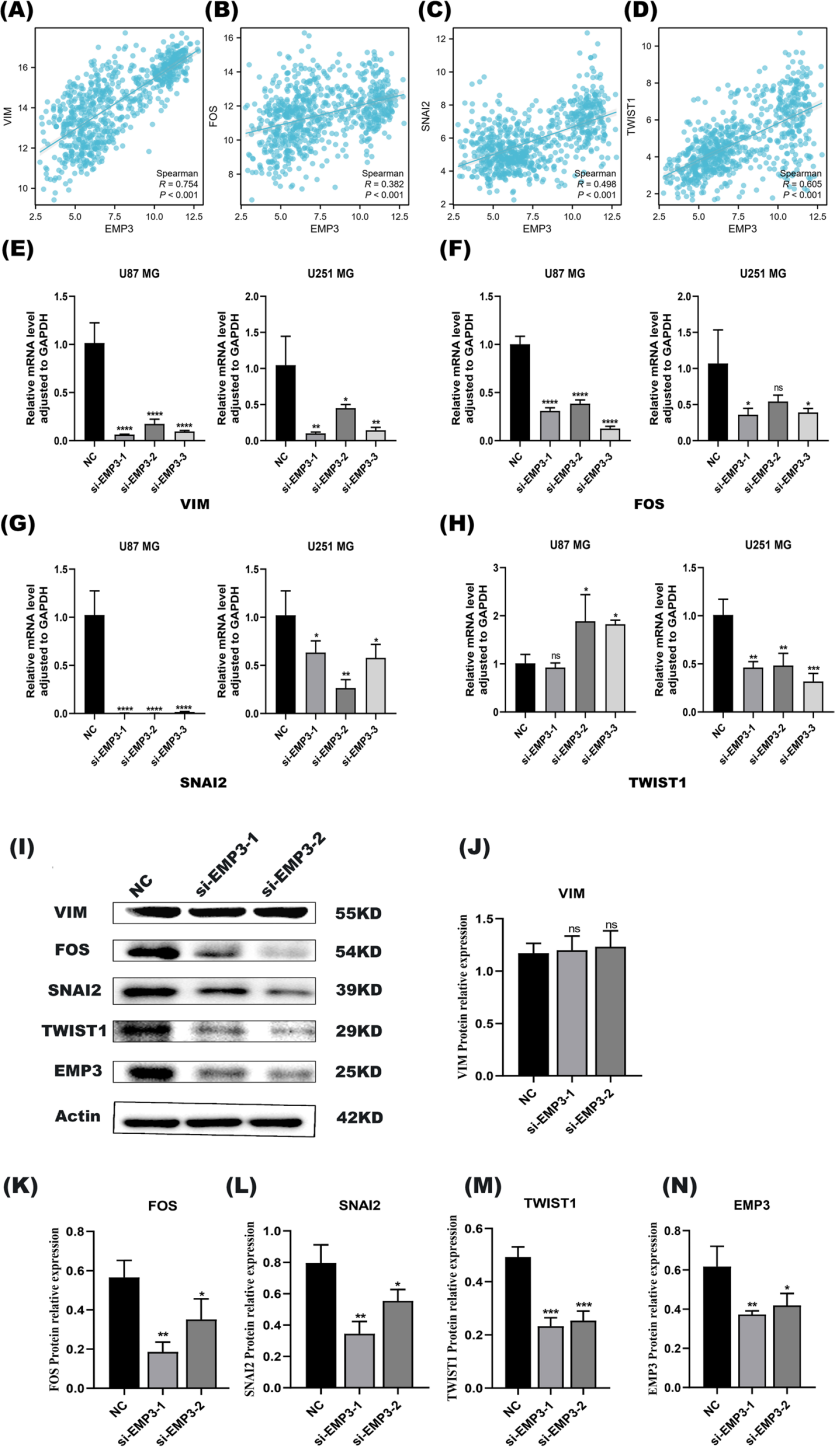
*EMP3* affects the malignant phenotype of glioblastoma by promoting the EMT process (**A**)-(**D**). Correlation analysis of *VIM, FOS, SNAI2, TWIST1* and *EMP3* using TCGA data (**E**)-(**H**). These related EMT markers were tested by qRT-PCR after the knockdown of *EMP3* for 96 h cultured with siRNA. On the other hand, proteins of EMT markers VIM, SNAI2, FOS, TWIST1 were investigated by western blot after *EMP3* siRNA transient transfection (**I**)-(**M**), Actin as the internal parameter. Cells were divided into three groups, NC (negtive control) group, siRNA *EMP3-1* and siRNA *EMP3-2* group. U87 cells were lysis after siRNA incubation for 72 h. The efficiency of trasfection of siRNA *EMP3* was detected by WB (**N**). All of the full-length blots/gels of western blot are presented in Supplementary Figure 1C-H. *p < 0.05, **P < 0.01, ***P < 0.001,****p < 0.0001

Epithelial-mesenchymal transition (EMT) is the process by which epithelial cells acquire mesenchymal cell characteristics. EMT is involved in tumourigenesis, invasion and metastasis and is a critical process in cancer progression. To determine the role of *EMP3* on EMT in glioblastoma, we first performed a correlation analysis of gene expression, which showed that *EMP3* was positively correlated with *VIM*, *FOS, SNAI2* and *TWIST1* (*p* < 0.001, R: *VIM*: 0.754, *FOS*:0.382, *SNAI2*: 0.498, *TWIST1*: 0.605, Fig. 6A-D). Next, at about 96 h by transient transfection of siRNA, we observed that the expression of *VIM*, a mesenchymal marker of EMT, and *SNAI2*, a transcription factor of EMT, were inhibited by silencing of *EMP3* (Fig. 6E, G). In U87 MG, *FOS* expression was significantly inhibited after silencing of *EMP3*, but in U251 MG, only the *si-EMP3-1* and *si-EMP3* groups were inhibited, with no significant difference between the *si-EMP3-2* group and the control group (Fig. 6F). Notably, *TWIST1* expression levels were significantly different between U87 MG and U251 MG. In U251 MG, *TWIST1* expression was suppressed; in contrast, in U87 MG, *TWIST1* expression was no significant difference was observed between the *si-EMP3* group and the control group (Fig. 6H). The decreased expression levels of *VIM, SNAI2*, and *FOS* after silencing *EMP3* suggest that *EMP3* may increase the malignant progression of tumors by promoting the EMT process. The difference in *TWIST1* expression may be due to tumor heterogeneity.

On the other hand, protein levels of EMT markers were investigated by western blot after *EMP3* siRNA transient transfection in U87 cells for 72 h. SNAI2, TWIST1 and FOS expression were significantly inhibited after silencing of EMP3 (Fig. 6I, K, L, and M), and in the *si-EMP3-1* and *si-EMP3-2* groups those proteins were inhibited, but with no significant difference between each other. The protein VIM expressed stably with no significant difference after *EMP3* siRNA transfection (Fig. 6J). The efficiency of trasfection of siRNA *EMP3* was detected by western blot (**N**).*p < 0.05, **P < 0.01, ***P < 0.001,****p < 0.0001 All of the full-length blots/gels are presented in Supplementary Figure 1C-H.

## Discussion

GBM is a high-grade cancer of glial cell origin with a poor prognosis. It is highly resistant to conventional treatments such as surgery, radiotherapy and chemotherapy, and surviving cells exhibit a more aggressive nature [23]. Patients with GBM often present with headaches, neurological deficits, seizures, nausea, vomiting, or other neurological sequelae. Many patients have a severe disease burden at the time of initial presentation and a poor prognosis, with a median survival of fewer than two years [24]. The current backbone of systemic treatment for GBM is temozolomide (TMZ). This alkylating agent targets the N-7 or O-6 position of guanine residues in DNA. the role of TMZ was established in the EORTC 26981/22981 and NCIC CE.3 trials, also known as the Stupp trial, which compared postoperative radiotherapy alone with postoperative radiotherapy and TMZ treatment followed by up to 6 cycles of TMZ maintenance treatment. Overall survival rates were significantly longer in the TMZ group, 27.2% at two years and 10% at five years, compared to 10.9% and 1.9% in the radiotherapy alone group [25]. The addition of TMZ has shown a survival benefit, particularly for patients with *MGMT* promoter methylation, which is present in approximately 50% of tumor patients [26]. As GBM is susceptible to tolerance to various treatment modalities, there is currently no clinically effective treatment for GBM. In recent years, new therapeutic strategies such as molecularly targeted therapies, immunotherapy and tumor electric field therapy have offered hope for highly drug-resistant GBM. Targeted therapies based on BRAFV600E mutation, NF1 mutation, EGFRvIII mutation and anti-angiogenesis are already available after preliminary clinical trials [27]. The discovery of new targets for developing precision-targeted therapy for GBM patients is now promising.

*EMP3* expression is upregulated in brain tumors, particularly in GBM. Ernst et al. [28] showed high *EMP3* levels in GBM spherical cultures. Scrideli et al. [29] also found higher expression of *EMP3* in GBM. *EMP3* mRNA expression has been shown to correlate with poor prognosis [30], and deletion of *EMP3* protein reduces malignant behavior in GBM cells [31]. Consistently with these previous findings, our single-cell analysis revealed *EMP3* correlates with EMT score in high-grade glioblastoma cells. We analyzed the expression levels of *EMP3* in different clinicopathological types of glioma based on mRNA-seq data from glioma patients in the CGGA and TCGA databases. Our results elucidate that *EMP3* is enriched in more malignant glioma subtypes and that univariate and multifactorial analyses show that *EMP3* expression is an independent risk factor for OS in glioma patients. Patients who has higher mRNA level of *EMP3* perform worse prognosis in clinical practice. Functional experiments showed that the knockdown of *EMP3* significantly suppressed the malignant phenotype of tumor cells in terms of proliferation and migration. Consistent with other literature reports, Anke Zhang et al. [32] demonstrated *EMP3* as a novel predictor for clinical progression and clinical outcomes in glioma. However, what are the mechanisms and reasons behind it? So far, there is still no clear indication. In our study, silencing of *EMP3* inhibits the malignant behavior of glioblastoma cells by regulating the EMT process mechanistically. GUO et al. [3] found that low expression of *EMP3* can regulate the EMT process, hinder its development, and thus inhibit the invasion of gastric cancer cells, which corresponds to our results. Overall, our results reveal that *EMP3* can predict poor prognosis in glioblastoma patients.

EMT process involves some fundamental processes, including embryonic evolution, tissue formation, wound healing and tissue fibrosis. In addition, EMT has been shown to promote tumor cell growth, drug resistance and proliferation. High-expression of mesenchymal gene signature predicts poor prognosis in glioma patients [33], suggesting that EMT-like processes are closely associated with GBM invasion. Several key signaling pathways, including transforming growth factor beta (TGF-β), Wnt, Notch and Hedgehog, are known to be involved in EMT [34]. These signaling pathways ultimately lead to the activation of EMT transcription factors (EMT-TFs). Several transcription factors have been identified as master regulators of EMT, including *SNAI2* factors, bHLH factors (E12 and E47, *TWIST1* and *TWIST2*) [35]. *SNAIL1/2* and *TWIST1/2* are considered the main regulatory factors driving the transcription pathway of EMT, and they converge to activate the expression of transcription factors [36]. *SNAIL1* and *SNAIL2* are involved in embryonic development, fibrosis, tumor development, and activation of EMT [37], and together with other transcriptional regulatory factors, they control gene expression. Besides, there are also newly identified transcription factors, which is a crucial regulator of tumor progression [38], and a critical transcription factor for EMT [39], such as *FOSL2*. In the SMAD dependent pathway, activation of the SMAD complex by the TGF-β receptor induces increased expression of the mesenchymal marker *VIM* [40]. Key transcription factors mediate the transition of cells from epithelial to mesenchymal transition, and these transcription factors mainly regulate the process intercellular adhesion, cell polarity, and vitality [41], and metabolism, transcription, differentiation. Transcription factors inhibit genes associated with epithelial phenotype induction of mesenchymal gene expression leads to EMT characteristics in cell [42]. Under specific physiological environments, the expression of SNAIL can be activated through various signaling pathways, including TGF-β, Wnt, Notch and growth factors acting on RTK [43] indicate that SNAIL related EMT processes are influenced by multiple mechanisms Control driven [44]. Related studies have found that down regulation of SNAI2 blocks the ability of TWIST to activate EMT in breast cells, suggesting that TWIST can indirectly induce E-cadherin transcriptional inhibition [45].

In our study, we found a positive correlation between *EMP3* and the expression of *VIM, FOS*, and *SNAI2* genes. Zhou W et al. found *SNAI2* increased the migration and invasion ability of tumors by promoting the loss of cell adhesion and polarity [46]. We found that SNAI2, TWIST1 and FOS protein expressions were lower in *EMP3* knockdown U87 cells than U87 wide type. This result implied that *EMP3* might promote EMT process. Interestingly, VIM did not perform the same change as others. VIM plays an integral role in lamellipodia formation and maintenance of cell polarity in migrating cells [47]. Dynamic reorganization of VIM might be continued in the lamellipodia for the formation of cellular polarity, without the influence of *EMP3*. However, the varying mRNA expression of *TWIST1* in U87 and U251 cell lines following *EMP3* knockdown could be attributed to the fact that EMT can be active in different regions of the tumor and at different stages of cancer progression. This could result in the involvement of different EMT-associated transcription factors (EMT-TFs) at distinct stages of tumor progression, leading to EMT heterogeneity. Therefore, based on our observations, it can be inferred that *EMP3* might be promoting the malignant phenotype of GBM via the EMT process. Nevertheless, the exact mechanism underlying this process warrants further investigation.

Whether it is related to the tumor microenvironment of immunosuppression? Recently, Qun Chen et al. reported that EMP3 was associated with immunosuppression in GBM. Elevated EMP3 in GBM areas was accompanied by high expression of PD-L1 and abundant M2 Tumor-Associated Macrophage (TAM) recruitment but a lake of T cell infiltration. They found that EMP3 was a potent protein in M2 TAM polarization and recruitment that impaired the ability of GBM cells to secrete CCL2 and TGF-β1. Furthermore, EMP3 suppressed T cell infiltration into GBM tumors by inhibiting the secretion of CXCL9 and CXCL10 by macrophages and led to an effective response to anti-PD1 therapy [48]. This opinion coincided with what some scholars considered, EMT is associated with cancer cell stemness, metastasis, chemotherapy resistance, and immune suppression [41, 49]. Tumor epithelial mesenchymal plasticity (EMP) refers to the transformation of tumor cells between epithelial like cells and completely or partially mesenchymal like cells [50, 51]. EMP is also a potential mediator causing resistance to immune checkpoint inhibitors [52, 53]. Currently, the precise role of *EMP3* in GBM remains not fully elucidated, and the exact in vivo function of *EMP3* calls for further verification through integrated spatial omics and ex vivo experimental investigations. The swift discernment of *EMP3's* functional mechanism could expedite the development of targeted therapies for GBM, and pave the way for discovering new therapeutic strategies to tackle chemoresistance, thereby improving clinical outcomes for GBM patients.

## Conclusion

In this study, we employed a combination of bioinformatics and experimental approaches to uncover that *EMP3* is highly expressed in GBM and correlates with inferior overall survival rates. Additionally, we identified *EMP3* as a potential oncogene tied to the EMT process. Given these findings, considering *EMP3* as a novel biomarker is of significant importance for early detection and therapeutic strategies in glioma patients. It is promising to envisage the potential survival advantages that could stem from targeting *EMP3* in GBM patients.

### Supplementary Information

**Additional file 1.** In Fig. 5, we checked proliferation and migration of GBM cells after we knocked down EMP3. We added the knockdown efficiency of EMP3 in glioma cells, which were detected by western blotting experiments (*p* < 0.01, Fig 5G). Cells were divided into four groups, NC (negtive control), siRNA EMP3-1, siRNA EMP3-2 and siRNA EMP3-3. U251 cells were lysis after siRNA incubation for 72 hours. All of the full-length blots/gels are presented in Supplementary Figure 1A and B. In Fig6, we detected the protein levels of EMT related factors after EMP3 siRNA transfection. Results of Western blotting indicated SNAI2, TWIST1 and FOS expression were significantly inhibited after silencing of EMP3 (Fig 6I, K, L, M). The protein Vim expressed stably with no significant difference after EMP3 siRNA transfection Fig 6J). The efficiency of trasfection of siRNA EMP3 was detected by western blot (N).*p <0.05, **P < 0.01, ***P < 0.001,****p <0.0001 All of the full-length blots/gels are presented in Supplementary Figure 1C-H.

**Additional file 2.** All of the full-length gels and blots are shown included in the Supplementary Information file named “Supplementary figure 1A-H. These images are original, unprocessed versions. Unfotunately, the molecular weight of the targeted proteins in this study are relatively close, so we can not detect them clearly in the same gel at the same time. Such as VIM and FOS, with molecular similar weights of 55KD and 54KD respectively, they were blotted separately, transferred, and incubated in batches. Similar to some other transcription factors of EMT in other literatures, separate gels were also used to test targeted proteins in the experiment (Lee J, You JH, Kim MS, Roh JL. Epigenetic reprogramming of epithelial-mesenchymal transition promotes ferroptosis of head and neck cancer. Redox Biol. 2020 Oct; 37:101697. doi: 10.1016/j.redox.2020.101697. Epub 2020 Aug 28. PMID: 32896720; PMCID: PMC7484553).

**Additional file 3.**

## Data Availability

All the bioinformatics analysis data on gene expression, survival analysis and ROC curve in glioblastoma in this paper were obtained from TCGA and CGGA. All data used in this study is available from the corresponding author on reasonable request.
