# Supplementary material for: EMP3 as a prognostic biomarker correlates with EMT in GBM
Source: BMC Cancer. 2024 Jan 17;24:89. doi: 10.1186/s12885-023-11796-0 (PMC10792875; doi:10.1186/s12885-023-11796-0)
Supplement: Supplementary file 1 — Additional file 1. In Fig. 5, we checked proliferation and migration of GBM cells after we knocked down EMP3. We added the knockdown efficiency of EMP3 in glioma cells, which were detected by western blotting experiments (p < 0.01, Fig 5G). Cells were divided into four groups, NC (negtive control), siRNA EMP3-1, siRNA EMP3-2 and siRNA EMP3-3. U251 cells were lysis after siRNA incubation for 72 hours. All of the full-length blots/gels are presented in Supplementary Figure 1A and B. In Fig6, we detected the protein levels of EMT related factors after EMP3 siRNA transfection. Results of Western blotting indicated SNAI2, TWIST1 and FOS expression were significantly inhibited after silencing of EMP3 (Fig 6I, K, L, M). The protein Vim expressed stably with no significant difference after EMP3 siRNA transfection Fig 6J). The efficiency of trasfection of siRNA EMP3 was detected by western blot (N).*p <0.05, **P < 0.01, ***P < 0.001,****p <0.0001 All of the full-length blots/gels are presented in Supplementary Figure 1C-H. [file 12885_2023_11796_MOESM1_ESM.doc]

1.Fig5(G)
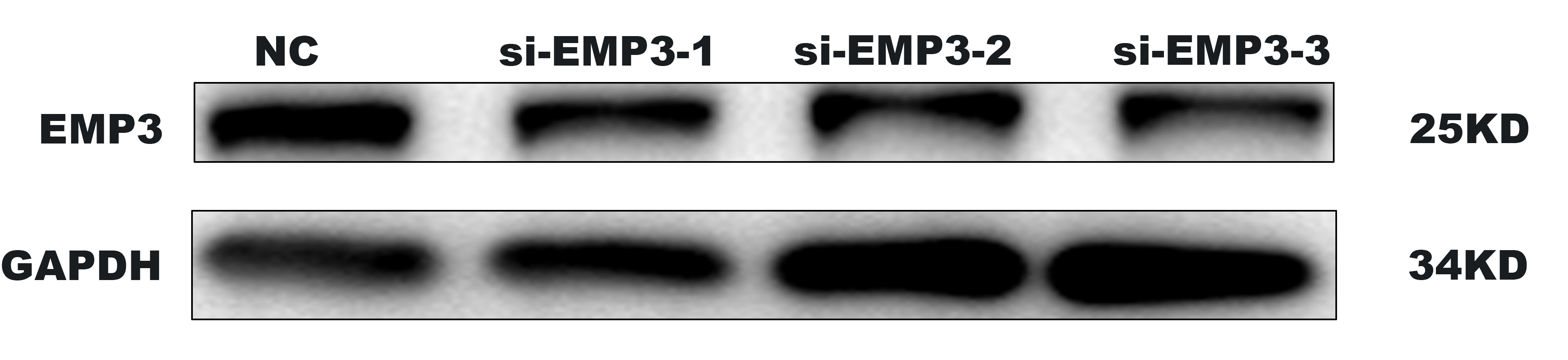


In Fig.5, we checked proliferation and migration of GBM cells after we knocked down EMP3. We added the knockdown efficiency of EMP3 in glioma cells, which were detected by western blotting experiments(p < 0.01, Fig 5G). Cells were divided into four groups, NC(negtive control), siRNA EMP3-1, siRNA EMP3-2 and siRNA EMP3-3. U251 cells were lysis after siRNA incubation for 72 hours. All of the full-length blots/gels are presented in Supplementary Figure-1 (A)and (B).

1. Fig6(I)-(N).


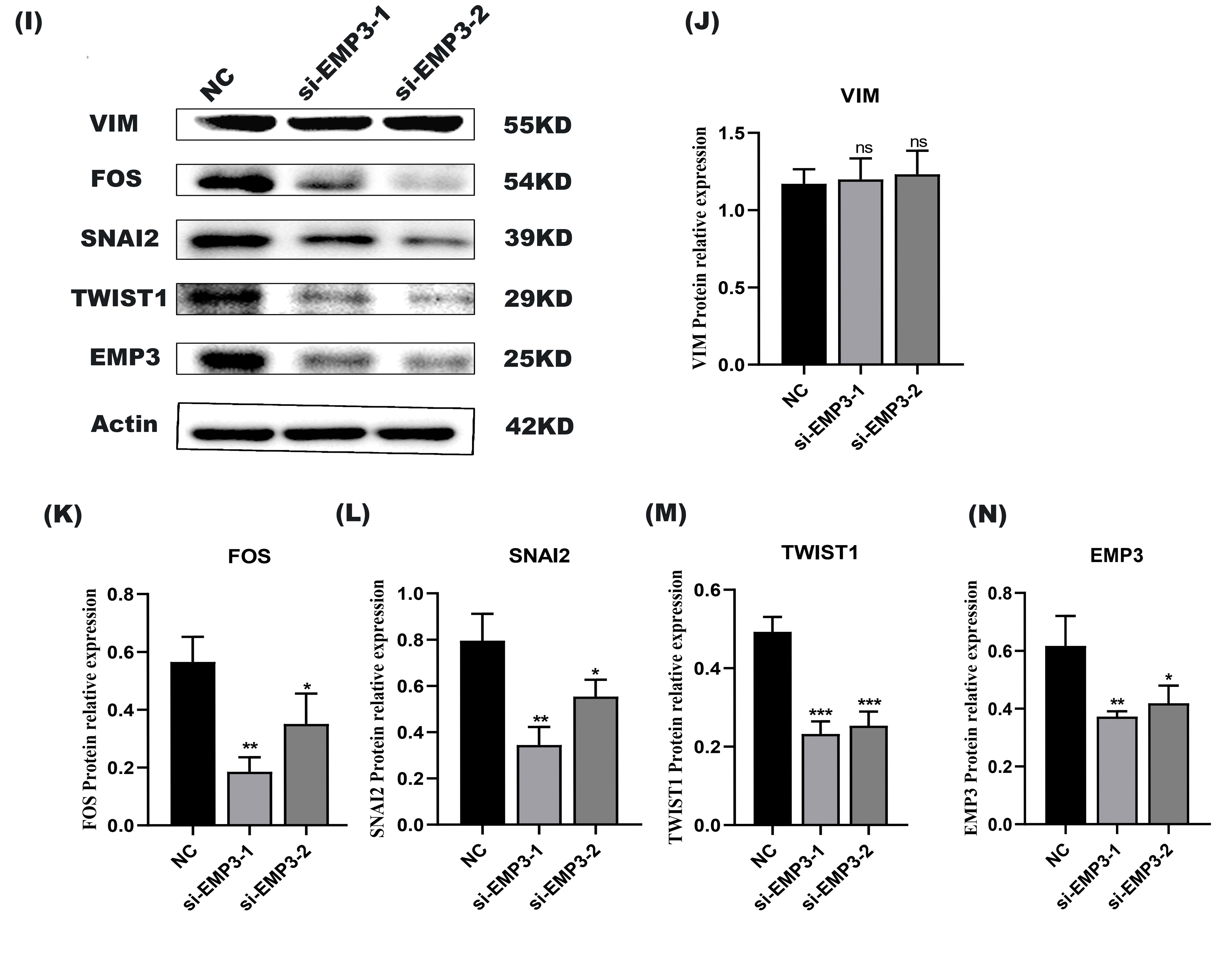


In Fig6, we detected the protein levels of EMT related factors after EMP3 siRNA transfection. Results of Western blotting indicated SNAI2, TWIST1 and FOS expression were significantly inhibited after silencing of EMP3(Fig 6I, 6K, 6L, 6M). The protein Vim expressed stably with no significant difference after EMP3 siRNA transfection(Fig 6J). The efficiency of trasfection of siRNA EMP3 was detected by western blot(N).*p<0.05, **P < 0.01, ***P < 0.001,****p<0.0001 All of the full-length blots/gels are presented in Supplementary Figure-1 (C)-(H).

1. In the introduction and discussion part of the article, abundant summary of the relevant background are placed with highlighted letters.
2. Some of the authors' employers changed because students went to work in hospitals after graduation.
3. Western blot methods were added in the manuscript.

**2.4 Western blotting(WB) analysis**

Protein was extracted from the cells were resolved by SDS-PAGE and then transferred to PVDF membranes (IPVH00010， Millipore), and then incubated with primary antibodies diluted in blocking buffer at 4 °C overnight. The following primary antibodies were used: Mouse Anti-β-Actin(HC201, TransGen Biotech, 1/2000), HRP conjugated Goat Anti-Mouse IgG (H+L) (GB23301, Servicebio, 1/2000), Rabbit Anti EMP3 (DF14661, Affinity, 1/1000), Rabbit Anti TWIST1(AF4009，Affinity，1/1000), Rabbit Anti SNAI2 (PB9439, Boster, 1/1000), Rabbit Anti FOS (AF5354，Affinity, 1/1000), Mouse Anti-VIM(60330-1-Ig, Proteintech,1/10000), HRP conjugated Goat Anti-Rabbit IgG (H+L)(GB23303, Servicebio, 1/2000), HRP conjugated Goat Anti-Mouse IgG (H+L) (GB23301, Servicebio, 1/2000)was used. Finally, the antigen-antibody reaction was visualized by the enhanced Pierce ECL Western blotting substrate kit (Thermo Scientific/ Pierce, Rockford, IL, USA).
