# Supplementary material for: EMP3 as a prognostic biomarker correlates with EMT in GBM
Source: BMC Cancer. 2024 Jan 17;24:89. doi: 10.1186/s12885-023-11796-0 (PMC10792875; doi:10.1186/s12885-023-11796-0)
Supplement: Supplementary file 2 — Additional file 2. All of the full-length gels and blots are shown included in the Supplementary Information file named “Supplementary figure 1A-H. These images are original, unprocessed versions. Unfotunately, the molecular weight of the targeted proteins in this study are relatively close, so we can not detect them clearly in the same gel at the same time. Such as VIM and FOS, with molecular similar weights of 55KD and 54KD respectively, they were blotted separately, transferred, and incubated in batches. Similar to some other transcription factors of EMT in other literatures, separate gels were also used to test targeted proteins in the experiment (Lee J, You JH, Kim MS, Roh JL. Epigenetic reprogramming of epithelial-mesenchymal transition promotes ferroptosis of head and neck cancer. Redox Biol. 2020 Oct; 37:101697. doi: 10.1016/j.redox.2020.101697. Epub 2020 Aug 28. PMID: 32896720; PMCID: PMC7484553). [file 12885_2023_11796_MOESM2_ESM.doc]

All of the full-length gels and blots are shown included in the Supplementary Information file named “Supplementary figure-1(A-H). These images are original, unprocessed versions. Unfotunately, the molecular weight of the targeted proteins in this study are relatively close, so we can not detect them clearly in the same gel at the same time. Such as VIM and FOS, with molecular similar weights of 55KD and 54KD respectively, they were blotted separately, transferred, and incubated in batches. Similar to some other transcription factors of EMT in other literatures, separate gels were also used to test targeted proteins in the experiment(Lee J, You JH, Kim MS, Roh JL. Epigenetic reprogramming of epithelial-mesenchymal transition promotes ferroptosis of head and neck cancer. Redox Biol. 2020 Oct;37:101697. doi: 10.1016/j.redox.2020.101697. Epub 2020 Aug 28. PMID: 32896720; PMCID: PMC7484553).
